# Understanding the podocyte immune responses in proteinuric kidney diseases: from pathogenesis to therapy

**DOI:** 10.3389/fimmu.2023.1335936

**Published:** 2024-01-15

**Authors:** Hong Jiang, Zhirang Shen, Jing Zhuang, Chen Lu, Yue Qu, Chengren Xu, Shufen Yang, Xuefei Tian

**Affiliations:** ^1^ Division of Nephrology, Department of Internal Medicine, People’s Hospital of Xinjiang Uygur Autonomous Region, Urumqi, China; ^2^ Division of Nephrology, Department of Internal Medicine, The First Affiliated Hospital of Xinjiang Medical University, Urumqi, China; ^3^ Section of Nephrology, Department of Internal Medicine, Yale University School of Medicine, New Haven, CT, United States

**Keywords:** podocyte, immunity response, pathogenesis, proteinuria, treatment

## Abstract

The glomerular filtration barrier, comprising the inner layer of capillary fenestrated endothelial cells, outermost podocytes, and the glomerular basement membrane between them, plays a pivotal role in kidney function. Podocytes, terminally differentiated epithelial cells, are challenging to regenerate once injured. They are essential for maintaining the integrity of the glomerular filtration barrier. Damage to podocytes, resulting from intrinsic or extrinsic factors, leads to proteinuria in the early stages and eventually progresses to chronic kidney disease (CKD). Immune-mediated podocyte injury is a primary pathogenic mechanism in proteinuric glomerular diseases, including minimal change disease, focal segmental glomerulosclerosis, membranous nephropathy, and lupus nephritis with podocyte involvement. An extensive body of evidence indicates that podocytes not only contribute significantly to the maintenance of the glomerular filtration barrier and serve as targets of immune responses but also exhibit immune cell-like characteristics, participating in both innate and adaptive immunity. They play a pivotal role in mediating glomerular injury and represent potential therapeutic targets for CKD. This review aims to systematically elucidate the mechanisms of podocyte immune injury in various podocyte lesions and provide an overview of recent advances in podocyte immunotherapy. It offers valuable insights for a deeper understanding of the role of podocytes in proteinuric glomerular diseases, and the identification of new therapeutic targets, and has significant implications for the future clinical diagnosis and treatment of podocyte-related disorders.

## 1 Introduction

The glomerulus, a specialized unit within the kidney, serves as a crucial filtration system that eliminates waste products from the blood. Maintaining internal stability by clearing external and internally produced waste is its primary function. Its filtration structure relies on the intricate network of glomerular capillaries, comprising glomerular endothelial cells, the glomerular basement membrane (GBM), and glomerular podocytes, collectively forming the unique filtration barrier of the glomerulus (1). Any impairment in this barrier may result in protein loss. Notably, proteinuric kidney disease originating from glomerular dysfunction accounts for 80% of individuals who eventually progress to end-stage kidney disease (ESKD), necessitating kidney replacement therapy (2).

Podocytes, the largest cells in the glomerulus, attach to the outer layer of the GBM. They consist of a cell body housing a nucleus, several prominent primary processes branching out from the cell, and secondary processes that further branch to form interdigitating foot processes (tertiary processes) on the GBM (3). The podocyte cytoskeleton plays a vital role in stabilizing its morphology and maintaining its biological function. It contributes to the integrity of the glomerular filtration barrier through interactions with cell-cell junctions and cell-matrix proteins (4). Podocytes can be categorized into three distinct membrane domains: the basal domain, the apical domain, and the intercellular/slit diaphragm domain. The slit diaphragm, a complex structure comprising multiple protein molecules, plays a pivotal role in regulating glomerular permeability, in coordination with the glomerular basement membrane and the glomerular endothelial cells. Key protein molecules within the slit diaphragm of podocytes include nephrin, neph1, neph2, CD2-associated protein(CD2AP), and podocin, among others (5). Furthermore, emerging evidence suggests that podocytes possess diverse functions beyond their mechanical supportive role, contributing to the innate and adaptive immune system to maintain glomerular health homeostasis (6). The immune malfunction of podocytes is considered to be associated with the pathogenesis of several common proteinuric glomerular diseases. Immune complexes, complement factors, various immune cells, and even the immune attributes of podocytes might contribute to the development of these proteinuric glomerular diseases. However, the intricate mechanisms of immune responses leading to podocyte injury remain incompletely understood. This review aims to consolidate current insights into the pathogenic mechanisms of immune responses triggering podocyte injury and the latest therapeutic strategies targeting the immune system.

## 2 Immunological mechanisms in podocyte

### 2.1 Innate immune responses in podocytes

An expanding body of evidence underscores the significant role of podocytes in the glomerular filtration barrier and their involvement in innate immune responses. Podocytes have been found to express various pattern recognition receptors (PRRs), including Toll-like Receptors (TLRs), enabling them to actively participate in innate immune responses by recognizing and eliminating foreign pathogens or endogenous danger signals (7). Notably, Toll-like Receptor 4 (TLR4) is a specific subtype capable of recognizing bacterial lipopolysaccharide (LPS) (8). Furthermore, exposure to a high-glucose milieu has been demonstrated to directly enhance the activation of TLR4. This activation is posited to contribute to the pathogenesis of podocyte damage and the subsequent progression of interstitial fibrosis (9). TLRs are present on the cell surface or intracellularly and are expressed by a range of cell types, including dendritic cells, macrophages, fibroblasts, B cells, T cells, as well as endothelial and epithelial cells (10). They play a pivotal role in recognizing pathogen-associated biomarkers (11). For instance, cell surface TLRs mainly recognize microbial membrane components like LPS, lipids, and proteins, while intracellular TLRs primarily detect bacterial and viral nucleic acids (12). However, it’s worth noting that some studies have reported that stimuli like puromycin aminonucleoside (PAN) can induce glomerular podocyte injury, increase Toll-like Receptor 9 (TLR9) expression, activate Nuclear factor kappa-B (NF-κB) and p38,mitogen-activated protein kinase (MAPK) signaling pathways, and potentially use endogenous mitochondrial DNA as a ligand. This results in damage to glomerular podocytes and leads to apoptosis, suggesting that TLRs can have a dual effect on podocytes—both protecting the integrity of the glomerular filtration barrier and potentially promoting glomerulosclerosis (13). Moreover, Masum and colleagues utilized BXSB/MpJ-Yaa (Yaa) mice to demonstrate that overexpression of TLR9 correlated with podocyte injury and the development of typical membranoproliferative glomerulonephritis (MPGN) lesions. In contrast, BXSB control mice, with weak TLR9 expression, showed no MPGN lesions. These findings suggest a potential role of TLR9 in podocyte injury and MPGN development (14).

**Figure 1:**
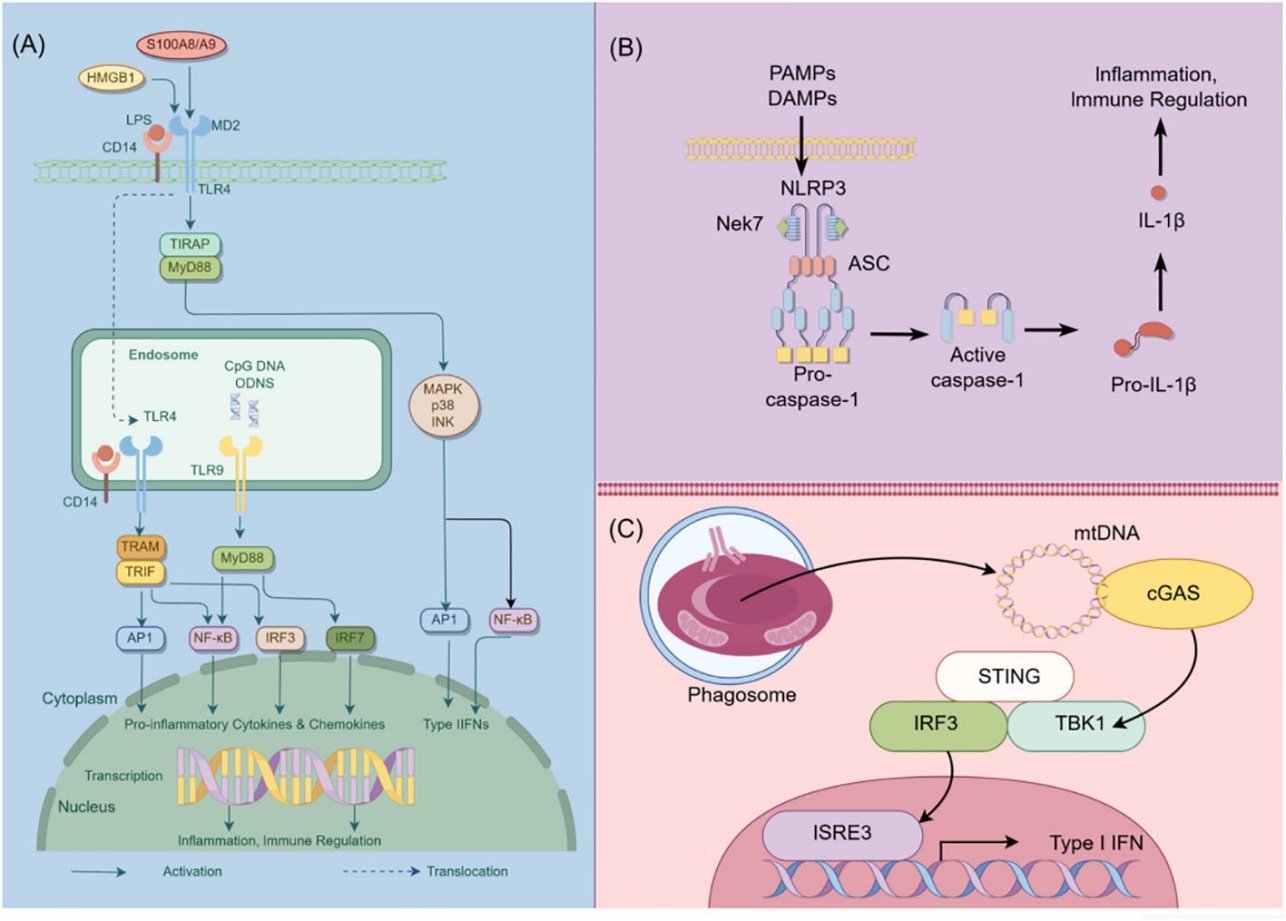
Schematic diagram of innate immunity in podocytes. This figure illustrates the role of TLR, NLRP3, and cGAS-STING signaling pathways in podocyte innate immunity. **(A)** Intracellular TLR signaling pathway: TLR, a pattern recognition receptor, can identify pathogen-related molecular patterns (PAMPs) and damage-related molecular patterns (DAMPs) within and outside cells, such as lipopolysaccharides (LPS). Upon TLR activation, downstream signaling molecules, including IRF3, IRF7, NF-κB, among others, are stimulated through various adapter proteins like MyD88, TRIF, etc., leading to the expression of antiviral and inflammatory genes. **(B)** NLRP3 Inflammasome activation: NLRP3, a member of the NOD-like receptor (NLR) family, is capable of recognizing intracellular PAMPs and DAMPS. Following NLRP3 activation, it assembles an inflammasome complex with ASC and Caspase-1, thereby triggering Caspase-1 activity and facilitating the maturation and secretion of IL-1β. **(C)** CGAS-STING signal pathway: CGAS, a cyclic GMP-AMP synthase, can detect DNA within cells. Upon eGAS binding to DNA, it synthesizes cyclic GMP-AMP (cGAMP) as a second messenger, which activates the STING protein. The STING protein subsequently triggers downstream signaling molecules like TBK1 and IRF3, leading to the expression of antiviral and inflammatory genes. HMGB1, High mobility group box-1 protein; LPS, Lipopolysaccharide; TIRAP, Toll-interleukin Receptor domain-containing adaptor protein; MyD88, Myeloid Differentiation Factor 88; MAPK, mitogen-activated protein kinases; AP1, activator protein-1; NF-κB, Nuclear factor kappa-B; IRF3, Interferon regulatory Factor 3; IRF7, Interferon regulatory Factor 7; PAMPs, pathogen-associated molecular patterns; DAMPs, damage-associated molecular patterns; NLRP3, NOD-like receptor thermal protein domain associated protein 3; Nek7, never in mitosis gene a-related kinase 7; ASC, apoptosis-associated speck-like protein containing a CARD domain; CGAS, cyclic guanosine monophosphate-adenosine monophosphate synthase; TBK1, TANK-binding kinase 1; STING, Stimulator of interferon genes; By Figdraw (www.figdraw.com).

In addition to TLRs, podocytes also express other critical PRRs that actively participate in processes related to inflammation, cell death, and proteinuria (15). For example, Nod-like receptors (NLRs) have been implicated in podocyte injury, with abnormal activation of the NOD-like receptor thermal protein domain associated protein 3 (NLRP3) inflammasome leading to podocyte damage (16). The activation of the NLRP3 inflammasome contributes to aldosterone-induced podocyte injury, yet studies indicate that its targeted silencing can substantially reduce the severity of such damage. This finding underscores the potential therapeutic role of NLRP3 inhibition in conditions characterized by aldosterone-mediated damage to podocytes (17). More recently, researchers have uncovered the significance of the Cyclic GMP-AMP synthase (cGAS) - Stimulator of interferon genes (STING) axis in podocyte innate immunity (18). cGAS serves as a PRR and is a key sensor of cytosolic Deoxyribonucleic acid (DNA) (19). It can catalyze the production of the second messenger 2’3’-cyclic GMP-AMP (cGAMP), which binds to STING, activates TANK binding kinase 1 (TBK1) and interferon regulatory factor 3 (IRF3), ultimately leading to the production of type I interferons (20). Upon activation, the cGAS-STING pathway triggers apoptosis or autophagy-mediated death in podocytes. This event initiates proteinuria and expedites the loss of podocytes (21). Consequently, these chain reactions contribute significantly to the acceleration of the development and the progression of glomerular diseases. The cGAS-STING pathway is a critical innate immune pathway that senses cytosolic DNA, contributing to antiviral immunity and inflammatory responses. High-mobility group box 1 (HMGB1), a nuclear protein that is overexpressed in podocytes, becomes actively secreted or passively released into the extracellular milieu following cellular damage (22). Upon its release, HMGB1 interacts with various receptors, notably the Receptor for Advanced Glycation End products (RAGE) and TLR4, which subsequently triggers podocyte apoptosis and promotes epithelial-mesenchymal transition (EMT)—processes that compromise podocyte integrity and function (23, 24). Therapeutic strategies that interrupt the HMGB1-receptor signaling axis have demonstrated potential in ameliorating podocyte injury, consequently enhancing the integrity of the glomerular filtration barrier (25). This avenue represents a promising therapeutic target for ameliorating the progression of CKD (**Figure 1**).

### 2.2 Adaptive immune responses in podocytes

**Figure 2:**
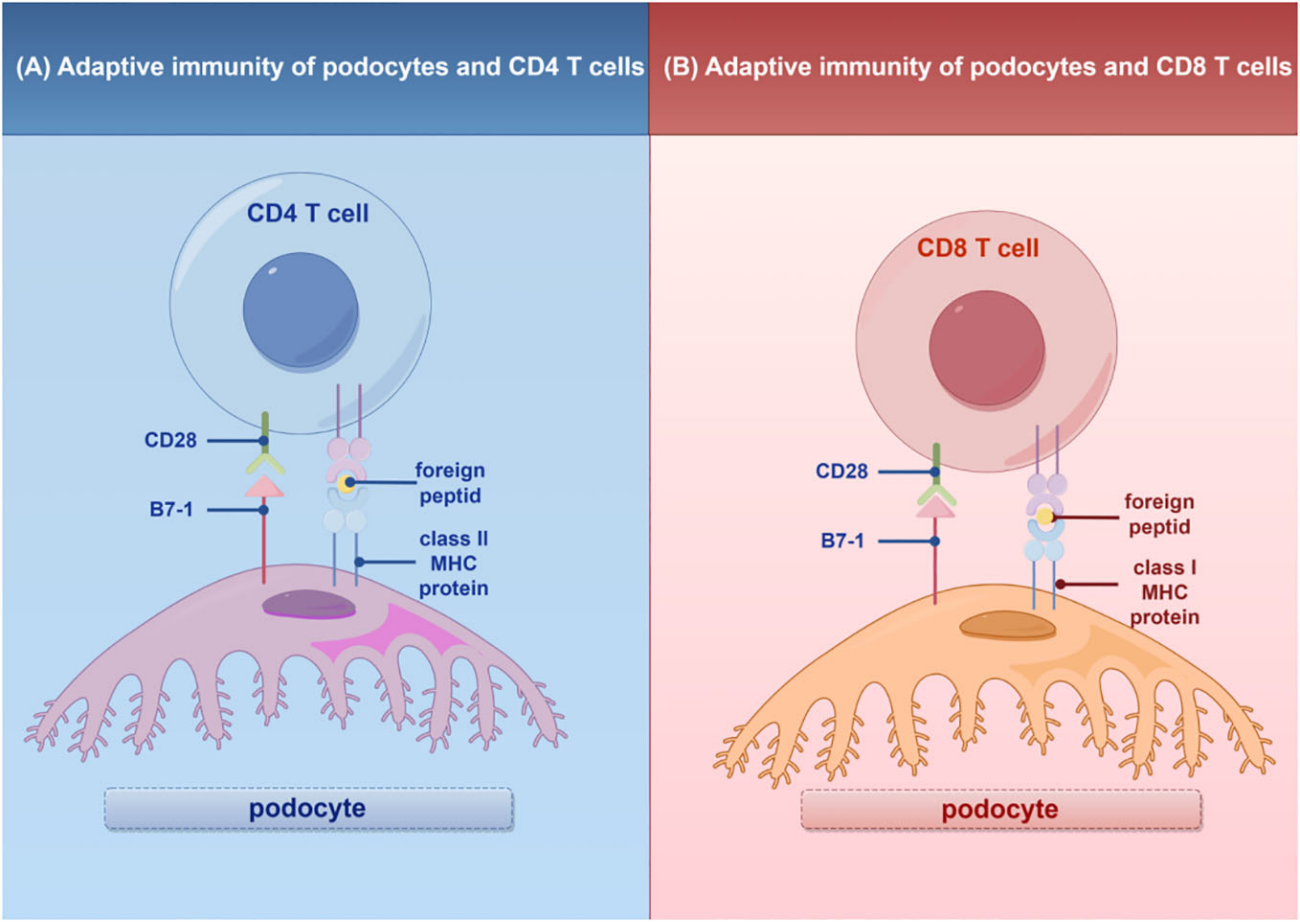
Adaptive immunity of T cells in podocytes. **(A)** Interaction of podocytes with CD4 T Cells: Podocytes express B7-1 and MHC II molecules, facilitating interactions with CD4 T cells and promoting glomerular inflammatory responses. **(B)** Interaction of podocytes with CD8 T Cells: Podocytes express B7-1 and MHC I molecules, enabling interactions with CD8 T cells and promoting glomerular inflammatory responses. MHC, major histocompatibility (www.figdraw.com). complex; By Figdraw.

**Figure 3:**
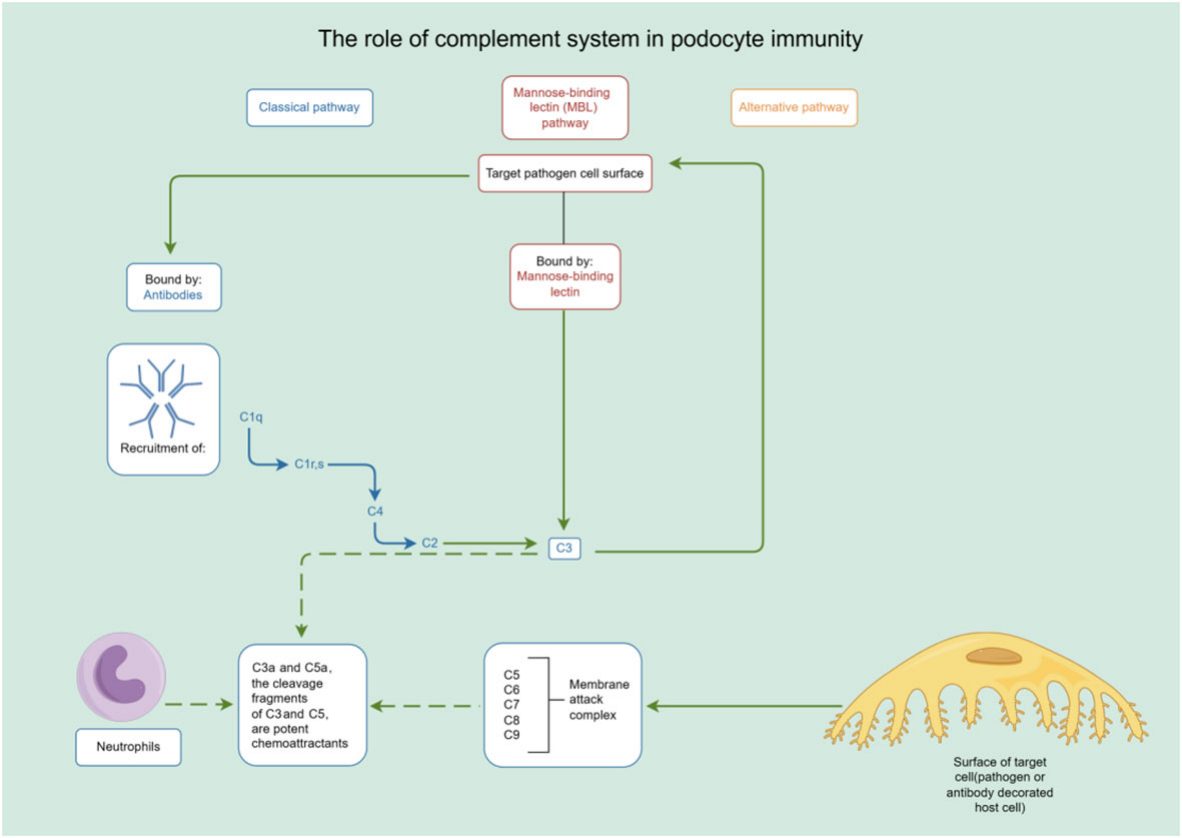
The role of complement system in podocyte immunity. Diagrammatic representation of complement system pathways and their involvement in podocyte immunity, highlighting the classical, lectin, and alternative pathways leading to pathogen lysis and immune cell recruitment. By Figdraw (www.figdraw.com).

In addition to their role in innate immunity, podocytes also participate in adaptive immune responses (26). Podocytes have the capacity to express major histocompatibility complex (MHC) class I and II antigens, which can respectively activate CD8^+^ and CD4^+^ T cells (27, 28). CD4^+^ T cells are involved in activating innate immune cells, B-lymphocytes, cytotoxic T cells, as well as nonimmune cells by secreting cytokines (29). On the other hand, CD8^+^ T cells exert specific cytotoxic effects, aiding in the body’s defense against pathogens and tumor cells (30). The activation of T cells relies on a two-signal process: the first signal involves the binding of MHC antigens to the T cell receptor (TCR), and the second signal entails the interaction between a co-stimulatory molecule and its ligand. Remarkably, podocytes can express B7-1 (CD80), which is a T cell co-stimulatory molecule that binds to CD28 on T cells, resulting in the generation of a positive synergistic stimulation signal. B7-1/CD80 is associated with B cells and antigen-presenting cells (APCs), and it can augment T cell responses to antigens presented by MHC class I/II (31). In contrast, B7-2 (CD86) is another co-stimulatory molecule found on B cells and APCs, which can also bind to CD28 (32) (**Figure 2**). These findings underscore the specific role of podocytes in adaptive immune regulation, which may have a significant impact on renal inflammation and injury. The complement system, an important integral component of innate and adaptive immune responses, has gained increasing attention in understanding the underlying pathogenesis of glomerular diseases. With a better understanding of the complement system and its role in glomerular diseases, treatments targeting complement activation are being explored for various glomerular conditions. Abnormal activation of the complement system is implicated in causing podocyte injury through multiple pathways. This involvement includes the production of reactive oxygen species (ROS), stimulation of cytokine release, and induction of endoplasmic reticulum stress. Collectively, these biochemical events compromise cytoskeletal stability, leading to podocyte detachment from the GBM and initiating the development of proteinuria (33). Studies investigating primary membranous nephropathy have demonstrated the critical involvement of the C3a/C3aR pathway in podocyte injury, noting that increased levels of plasma C3a and glomerular C3aR are associated with disease progression and serve as predictive indicators for patient prognosis (34). Podocytes serve a dual role in the narrative of complement-mediated damage: they are not only a target but also a source of complement system activation (35). Podocytes employ multiple defense mechanisms, including the expression of regulatory proteins like complement factor H and surface regulators such as CD46, CD55, and CD59, as well as processes like autophagy and actin-mediated endocytosis, to protect against and maintain homeostasis in the face of complement-induced damage (36). The interplay between the complement system and podocyte damage is intricate and comprises multiple dimensions. Elucidating the nuances of this relationship is pivotal for the innovation of therapeutic approaches aimed at treating conditions linked to podocyte damage (**Table 1**) (**Figure 3**).

**Table 1:** The role of immunity in podocyte injury.

Immunization route	Mechanism of action on podocytes	Reference
TLRs	TLRs recognize and eliminate foreign pathogens or endogenous danger signals. They can also induce glomerular podocyte injury, increase TLR9 expression, activate NF-κB and MAPK signaling pathways, and potentially use endogenous mitochondrial DNA as a ligand, leading to podocyte damage and apoptosis	(11, 13, 14, 37)
NLRs	Abnormal activation of the NLRP3 inflammasome leads to podocyte damage. NLRs actively participate in processes related to inflammation, cell death, and proteinuria	(38–41)
cGAS-STING	Upon activation, the cGAS-STING pathway triggers apoptosis or autophagy-mediated death in podocytes, initiating proteinuria and expediting the loss of podocytes	(18, 42)
HMGB1	HMGB1 interacts with various receptors, notably the Receptor for Advanced Glycation End products (RAGE) and TLR4, which subsequently triggers podocyte apoptosis and promotes epithelial-mesenchymal transition (EMT), compromising podocyte integrity and function	(24, 43, 44)
MHC class I and II antigens	MHC class I and II antigens can respectively activate CD8+ and CD4+ T cells, which are involved in activating innate immune cells, B-lymphocytes, cytotoxic T cells, as well as nonimmune cells by secreting cytokines	(27–29)
Complement system	The complement system is implicated in causing podocyte injury via multiple pathways, including the production of reactive oxygen species (ROS), stimulation of cytokine release, and induction of endoplasmic reticulum stress. This compromises the cytoskeletal stability, precipitating podocyte detachment from the GBM, and initiates the development of proteinuria	(33, 36)

## 3 Crosstalk between podocytes and other cells in the glomerulus

Cellular crosstalk is a fundamental process of intercellular signaling and interaction that exerts a significant influence on the behavior and function of various cells. This dynamic communication can occur between identical cell types or across different cell types (45). The interaction between other cells in the glomerulus and podocytes is critical in the development of glomerular diseases.Research has demonstrated a correlation between mitochondrial oxidative stress in glomerular endothelial cells and an increased expression of Endothelin-1 (Edn1) along with its receptor, Endothelin-1 receptor A (Ednra) (46). Targeted attenuation of mitochondrial oxidative stress or the disruption of the Edn1/Ednra signaling cascade has been shown to effectively ameliorate damage to glomerular endothelial cells, reduce the depletion of podocytes, decrease proteinuria levels, and attenuate the progression of glomerulosclerosis in D2 mice (47).

Vascular endothelial growth factor A (VEGF-A) and angiopoietin 1 (Ang-1) secreted by podocytes serve as protective factors that uphold the integrity and stability of endothelial cells by binding to receptors on the endothelial cells (48–50), Nevertheless, both the overexpression and deficiency of VEGF-A can lead to endothelial cell injury and proteinuria (51). Angiopoietin 2 (Ang2), primarily produced by glomerular endothelial cells, exhibits an antagonistic effect. It competitively inhibits the activation of the Tie2 receptor by Ang1, thereby increasing endothelial cell apoptosis and permeability (47). Additionally, vascular endothelial growth factor B (VEGF-B) secreted by podocytes acts as a pathogenic factor, fostering lipid accumulation and lipotoxicity, damaging the endothelial cell surface, and inducing proteinuria by binding to receptors on the endothelial cells (52).

Glomerular endothelial cells secrete endothelin-1 (ET-1), a vasoconstrictive peptide with multifaceted effects. ET-1 exerts diverse physiological and pathophysiological roles by binding to ETAR or ETBR on the surface of endothelial cells, mesangial cells, or surrounding cells (53). While ETAR primarily mediates vasoconstriction, cell proliferation, fibrosis, podocyte injury, and inflammatory response (54), ETBR predominantly mediates vasodilation, anti-proliferation, and anti-fibrosis, thereby exerting a protective effect (55). These crosstalk mechanisms significantly contribute to the regulatory processes involved in glomerular injury and repair.

Furthermore, the transforming growth factor β (TGF-β) released by podocytes activates various downstream signals within the Smad signaling pathway by binding to distinct receptors. This activation promotes mesangial cell proliferation, differentiation, and matrix deposition, influencing the phenotype and function of endothelial cells, mesangial cells, and podocytes themselves, ultimately leading to glomerular fibrosis (56). Exosomes, small extracellular vesicles, possess the capability to transport numerous molecular components, including proteins, lipids, DNA, miRNA, and lncRNA (57). Recent evidence has shed light on the role of exosomes in compromising the structural integrity of glomeruli, renal tubules, and renal interstitium. Under hyperglycemic conditions, mesangial cell-derived exosomes can potentially alter the functionality of podocytes through the delivery of TGF-β1, thereby contributing to the pathogenesis of diabetic nephropathy (58, 59).

Cytokines, such as TNF-α and TGF-β, secreted by mesangial cells, are known to reduce the expression of podocyte-associated proteins, including nephrin and podocin. This reduction weakens or disrupts junctions within the slit diaphragm, subsequently increasing the permeability of the glomerular filtration membrane. Consequently, proteinuria and impaired renal function ensue (60). This mechanism may partially elucidate the pathogenesis of FSGS.

The interplay between podocytes and other cells in glomeruli, as well as among podocytes themselves, plays a critical role in the pathogenesis of various kidney diseases, including glomerulonephritis, FSGS, and LN. This intricate process involves the complex interaction and regulation of various cytokines and receptors, ultimately contributing to the progression of glomerular damage and dysfunction. Understanding the molecular mechanisms underlying these crosstalks is instrumental in deciphering the pathogenesis of kidney diseases and serves as a foundation for the development of novel therapeutic strategies aimed at preserving glomerular function and ameliorating the progression of proteinuric glomerular diseases.

## 4 Common proteinuric glomerular diseases associated with immune responses in podocytes

### 4.1 Minimal change disease

MCD is the predominant type of nephrotic syndrome in children and adolescents. However, in adults, MCD accounts for a smaller proportion, approximately 10% to 16% of nephrotic syndrome cases (61). Clinically, MCD is characterized by massive proteinuria, hypoalbuminemia, edema, and dyslipidemia. Kidney biopsy typically reveals predominantly normal glomeruli under light microscopy, with the only notable finding being the diffuse disappearance of podocyte foot processes observed under transmission electron microscopy (62). Although the precise pathogenesis of MCD remains elusive, numerous research findings suggest its association with immune system dysregulation.

Traditionally, it has been postulated that MCD is mediated by certain unidentified circulating factors, possibly released by T cells, which target podocytes directly, leading to their dysfunction and consequent proteinuria (63). Evidence indicates that the circulating factors responsible for MCD may include specific cytokines, primarily belonging to the T helper 2 cell (Th2) subgroup, such as interleukin-13 (IL-13), interleukin-8 (IL-8), interleukin-4 (IL-4), among others (64–67). On one hand, T cell effector factors serve as the source of the aforementioned pro-inflammatory cytokines and others, which can directly or indirectly impair podocytes, resulting in proteinuria. On the other hand, regulatory T cells (Treg cells) maintain immune balance by binding to CD80 on antigen-presenting cells (APCs) through the expression of Cytotoxic T lymphocyte associate protein-4 (CTLA-4) (68). The reduced number and function of Treg cells in MCD patients may lead to immune dysregulation and an inflammatory response (69). Additionally, MCD has been associated with certain Treg cell-related genetic or acquired immune deficiency syndromes (70).

While much of the research on MCD has traditionally focused on T cell-related cytokines, recent studies have shed light on the involvement of B cells and specific autoantibodies, such as those targeting nephrin, a critical podocyte protein (71). The role of B cells in MCD pathogenesis has gained increased attention in recent years, particularly following the use of rituximab (RTX), a monoclonal antibody targeting the pan-B-cell marker CD20, which has shown efficacy in reducing recurrence rates (72, 73). In the process of antibody synthesis, B cells are capable of exerting a direct impact on the structural integrity and functional capacities of podocytes through the release of particular cytokines. Additionally, B cells can modulate T cell responses, thereby contributing to the pathogenesis of diseases. This dual capacity underscores the complex and integral participation of B cells in disease pathology, extending their influence beyond the classical role of antibody generation (74). Some studies suggest that B cells may contribute to MCD development by promoting T cell responses or producing autoantibodies. For instance, elevated levels of B cell-activating factor in the serum of MCD patients have been observed, which may drive T cell activation in MCD (75). Research has also revealed increased levels of T cells bearing immunoglobulin M (IgM) on their surface in patients with idiopathic nephrotic syndrome (INS) who exhibit poor treatment responses. *In vitro* experiments have demonstrated that these T cells can induce rearrangement of the podocyte cytoskeleton, indicating that the interplay between B cells and T cells may play a crucial role in MCD pathogenesis (76). Furthermore, IgM can trigger the classical complement activation pathway in the glomeruli of INS patients (77). These studies suggest that B cells may contribute to MCD through various mechanisms, but further research is required to elucidate their exact roles. In addition, certain autoantibodies have been considered mediators of MCD. Studies have shown that around 50% of children with relapsing hormone-sensitive nephrotic syndrome have elevated plasma anti-UCHL1 (ubiquitin C-terminal hydrolase-L1) antibody levels. However, in adult MCD patients, no elevation of anti-UCHL1 antibody levels was observed (78). This suggests the existence of different immune mechanisms in children and adults with MCD.

In summary, the pathogenesis of MCD involves complex interactions among the immune system, glomerular cells, and genetics, with multiple potential pathogenic factors and mechanisms at play. MCD is not a straightforward immune or podocyte disease. Heterogeneity and the absence of reliable animal models pose challenges to our understanding of MCD. Therefore, future research should focus on developing new experimental models, screening for new autoantibodies, and improving our understanding of cell-cell interactions in this disease.

### 4.2 Focal segmental glomerulosclerosis

FSGS is a group of glomerular lesions that were once considered to have a low incidence but have shown a gradual increase in global prevalence in recent decades. Reports suggest that the annual incidence of FSGS ranges from 0.2 to 1.8 cases per 100,000 people (79). Histopathologically, FSGS is characterized by partial sclerosis of some glomeruli, with each affected glomerulus showing only segmental involvement. Clinically, FSGS commonly presents as proteinuria, nephrotic syndrome, with or without kidney function impairment (80). While the precise mechanisms underlying the development of FSGS remain incompletely understood, emerging evidence suggests significant involvement of the immune system in the pathogenesis, with podocyte injury considered to be at the core of the disease.

FSGS has been linked to various immune-related factors, including infections, tumors, drugs, and genetic mutations, which can impact the structure and function of podocytes, leading to proteinuria and a decline in kidney function (81–85). T cells, a crucial component of the adaptive immune system, secrete various cytokines that regulate glomerular permeability and inflammatory responses. As early as 1974, Shalhoub proposed the hypothesis that FSGS is a T-cell-mediated disease (86). Subsequent research has identified abnormalities in different subtypes and functional states of T cells in FSGS patients. Some studies have reported a progressive decrease in the relative numbers of CD4 and CD8 T cells with disease progression (87). Reports have also demonstrated an increase in Th17 cells and a decrease in Treg cells among CD4 T cells in some adult patients with INS (88). Furthermore, various observations have associated the levels of certain cytokines secreted by T cells with glomerulosclerosis in FSGS patients. For example, interleukin-17 (IL-17), produced by the Th17 subgroup, has been linked to glomerulosclerosis in FSGS patients (89). Other studies have revealed that FSGS is related to the activation of the tumor necrosis factor-α (TNF-α) pathway in podocytes (90, 91). These cytokines may induce morphological changes, dedifferentiation, and apoptosis in podocytes by directly or indirectly affecting them. Podocytes express various TLRs involved in innate immunity, capable of activating signaling pathways that lead to morphological changes in podocytes and induce the expression of chemokines (92, 93). Moreover, TLR signaling can enhance the expression of CD80 on podocytes, a molecule that stimulates T cells (93, 94). Therefore, TLRs may also modulate the interaction between podocytes and T cells.

B cells are another crucial component of the adaptive immune system, primarily responsible for mediating humoral immune responses through the production of antibodies. Additionally, they perform various other immune functions. B cells function as efficient APCs, processing and presenting antigens to T cells, thereby activating T cell responses to antigens. Moreover, B cells secrete various cytokines, including both pro-inflammatory and anti-inflammatory factors, to regulate the balance of immune responses (95). While FSGS is not typically considered a typical immune complex-mediated disease, increasing evidence suggests that B cells also play a significant role in FSGS. Several potential autoantigens and antibodies associated with B cells have been identified in FSGS. For instance, annexin A2, a protein involved in apoptosis and signal transduction, has been identified as a potential autoantigen through mass spectrometry analysis (96). Another study found that 29% of INS patients had anti-nephrin immunoglobulin G (IgG) antibodies in their plasma, which decreased with treatment and disappeared in patients with complete remission (97). A recent report by Dr. Hattori and colleagues showed that circulating anti-nephrin autoantibodies detected in plasma samples from a recurrent FSGS patient after kidney transplantation results in the tyrosine phosphorylation of nephrin, leading to alterations in nephrin distribution and podocyte foot process effacement. It was indicated that circulating anti-nephrin autoantibodies could be a possible pathogenic candidate for circulating factors in the posttransplant recurrence of primary FSGS (97). Furthermore, there is evidence indicating a higher frequency of the human leukocyte antigen (HLA)-A30 antigen in primary FSGS patients, with HLA-A30 being associated with FSGS recurrence (98).

The Src homology 3 domain-binding protein 2 (SH3BP2) is an adaptor protein ubiquitously expressed in immune cells, governing signaling pathways within the cell (99). Kidney biopsy samples from patients with these conditions consistently exhibit upregulation of the SH3BP2 signaling complex, suggesting its potential involvement in immune activation—a prominent feature of these podocytopathies (100). Research led by Tarak Srivastava and colleagues, using murine models, has further underscored the significance of SH3BP2 in podocyte injury. Transgenic mice with induced mutations in the SH3BP2 gene displayed features indicative of podocyte dysfunction, including elevated urinary albumin levels, a decrease in serum albumin, and an increase in mesangial cell proliferation (100). Collectively, these findings position SH3BP2 and its associated proteins at the forefront of immunological processes that contribute to podocyte injury.

Despite the unclear pathogenesis of FSGS, comprehensively studying the structure and function of proteins related to podocytes, as well as the mechanisms underlying podocyte injury in FSGS, offers essential insights for deciphering its pathophysiology. These investigations could reveal novel avenues for clinical treatment, potentially resulting in the effective reduction or deceleration of the glomerulosclerosis process and postponing the onset of end-stage kidney failure.

### 4.3 Primary membranous nephropathy

Membranous nephropathy (MN) is a common kidney disease affecting individuals across different age groups, particularly adults. Pathologically, it involves the deposition of immune complexes on the outer side of the GBM located beneath the podocyte, accompanied by diffuse GBM thickening. Approximately 20% to 30% of MN cases are associated with secondary factors such as systemic autoimmune diseases, infections, drugs, malignancy, or hematopoietic stem cell transplantation, known as secondary MN. In cases where no clear causal relationship or recognizable systemic disease, infection, or drug exposure is identified, the condition is termed primary membranous nephropathy (pMN), often perceived as a kidney-specific autoimmune disease (101, 102). pMN is the most common cause of primary nephrotic syndrome in non-diabetic patients, accounting for approximately 30% of adult non-diabetic nephrotic syndrome cases. It is characterized by massive proteinuria, hypoalbuminemia, dyslipidemia, and edema, while a small number of patients present with asymptomatic proteinuria (103). The typical microscopic pathological features of pMN include GBM thickening, podocyte foot process effacement, and spike-like electron-dense deposits under the glomerular podocytes. Immunofluorescence analysis typically shows granular deposits of IgG and C3 along the capillary wall, with IgG4 being the dominant IgG subtype in pMN (104).

The pathogenesis of pMN is not yet fully elucidated. According to the classical theory, antigens on the podocytes bind to specific antibodies, forming *in situ* immune complexes that deposit at the GBM. This deposition activates complement and creates membrane attack complexes, leading to damage to the glomerular filtration barrier and subsequent proteinuria (105). Recent progress in understanding the pathogenesis of pMN has been driven by the discovery of two key podocyte antigens: phospholipase A2 receptor (PLA2R) and thrombospondin type-1 domain-containing 7A (THSD7A), which serve as antibody markers in 70% and 2-3% of pMN patients, respectively (106). Animal models have validated the pathogenicity of human PLA2R and THSD7A antibodies. Both PLA2R and THSD7A are transmembrane proteins expressed by podocytes that can bind to circulating IgG4 subtype autoantibodies, forming subepithelial immune complexes (107, 108). Tomas NM and colleagues engineered a transgenic mouse model that specifically expresses human phospholipase A2 receptor 1 (hPLA2R1) in podocytes. These mice spontaneously generate antibodies against hPLA2R1, resulting in the development of nephrotic syndrome and demonstrating classical histological features of MN (109). This rodent model faithfully recapitulates key aspects of human MN, providing a valuable tool for probing the pathophysiological underpinnings of the disease and for the assessment of targeted therapeutic interventions. Additionally, several other podocyte-intrinsic antigens, such as high-temperature requirement A1 (HTRA1), netrin G1 (NTNG1), contactin-1 (CNTN1), and Semaphorin 3B (SEMA3B), have been identified, although the pathogenicity of these antigens in MN remains to be confirmed (110–113).

Furthermore, some potential antigens that are not expressed by podocytes have been associated with pMN. Studies have shown that certain pMN cases are linked to the accumulation of exostosin glycosyltransferase 1 (EXT1) and exostosin glycosyltransferase 2 (EXT2). In a study of 48 PLA2R-negative pMN cases, EXT1 and EXT2 were detected in 21 of them but not in PLA2R-related pMN cases or control cases (114). Additionally, a mass spectrometry study conducted on 126 PLA2R-negative MN cases revealed that 29 cases were positive for neural epidermal growth factor-like 1 protein (NELL-1) (115). The clinical and kidney biopsy results of NELL-1-positive MN exhibited characteristics of pMN. In 5.7% of PLA2R-negative pMN cases, a unique protein called protocadherin 7 (PCDH7) was detected, and notably, all other antigens were negative in these cases (116). PCDH7-associated pMN appears to be a distinct, previously unidentified type of MN. Additionally, researchers have identified a novel antigen, neural cell adhesion molecule 1 (NCAM1), in rare pMN cases, with a prevalence of 2.0% in pMN cases (117). These antigens may be neo- or allo-antigens that deposit in the subepithelial space and bind to IgG1 or IgG3 subtype autoantibodies. The mechanism of action of these antigens in MN is unclear, and their potential associations with other diseases remain uncertain (102).

The pathogenesis of pMN is highly complex and results from the interaction of multiple factors. As our understanding of the molecular structure and gene loci of target antigens continues to grow, different target antigens and gene loci may provide new insights for the diagnosis and treatment of this disease. Further exploration of the mechanism of podocyte immunity in MN and the discovery of more effective and safe therapeutic targets are crucial directions for future pMN research.

### 4.4 Lupus nephritis with podocyte injury

Systemic lupus erythematosus (SLE) is a complex autoimmune disease that affects multiple systems and organs throughout the body. It’s estimated that up to 40% of SLE patients will develop lupus nephritis (LN) (118). LN is a common and serious complication of SLE, significantly impacting the long-term prognosis of patients. The hallmark histopathological feature of LN is the deposition of immune complexes and inflammatory reactions in the kidneys. This ultimately leads to the destruction of kidney parenchyma and a clinical decline in kidney function (119). The most internationally recognized pathological classification of LN is the 2003 classification of lupus nephritis by the International Society of Nephrology (ISN) and the Renal Pathology Society (RPS), which divides LN into types I-VI (120). However, this classification system does not encompass all types of LN pathology. Some LN patients may have kidney biopsy results that do not align with their clinical manifestations, such as having massive proteinuria with relatively normal glomeruli or only mild glomerular mesangial proliferation. These patients may have a unique subtype of LN known as LN with podocyte lesions (119).

The pathogenesis of LN is not completely understood, but the prevailing view is that LN is characterized by the deposition of immune complexes (IC) formed by various autoantibodies, such as anti-double-stranded DNA (ds-DNA) antibodies and antigens. Infiltration of inflammatory cells occurs, with podocytes being one of the primary targets of IC attacks (121). Anti-dsDNA antibodies can recognize various DNA structures and also cross-react with various non-DNA molecules present in the renal matrix or on the surfaces of endogenous renal cells, such as α-actinin, annexin II, collagen III/IV, and others (122–124). Additionally, the degree of glycosylation of anti-dsDNA antibodies can affect their pathogenicity, with α-2,6-sialylation attenuating their effects and fructosylation exacerbating their nephritogenic activity (125). The deposition of IC can activate the complement system, leading to the production of various complement components, such as C3, C4, C5a, and more. These complement components can attract and activate inflammatory cells like neutrophils, monocytes, and macrophages, which in turn release podocyte-toxic substances, resulting in direct damage or apoptosis of podocytes (126). An alternative hypothesis posits that the interplay of podocytes and TLRs could be a contributing factor in the pathogenesis of LN. The overexpression of TLR8 and TLR9 plays a key role in stimulating the NF-κB pathway, which results in the generation of specific inflammatory cytokines, namely IL1b, IL6, IFN-g, and TNF-α (127). This, in turn, intensifies the inflammatory response and contributes to glomerular damage. Significantly, an increase in TLR9 expression has been robustly associated with podocyte impairment. This is evident in the decline in podocyte numbers, loss of foot processes, and emergence of microvillous protrusions, along with the thickening and subsequent wrinkling of the basal membrane (14).

Recent research has revealed that, in addition to the involvement of relevant aberrant autoantibodies in antigen-antibody reactions contributing to the pathogenesis of LN, renal cells actively participate in the inflammation and immune response of LN. Kidneys harbor tertiary lymphoid structures (TLSs), resembling lymph node-like structures housing immune cells such as T cells, B cells, dendritic cells, macrophages, and more. TLSs serve as local immunity structures that promote adaptive immunity; their unencapsulated structure enables direct exposure to diverse stimuli from an inflamed environment, including CKD. These immune cells proliferate, differentiate, activate, and form memory within TLSs, actively engaging in the production of autoantibodies and pro-inflammatory factors, ultimately causing kidney tissue damage. Functional characterization of TLSs in CKD and the development of interventions to regulate kidney TLSs potentially lead to promising therapeutic avenues (128, 129). Notably, the preferential expression of TCR Vβ gene expression in intrarenal T cells, driven by antigen stimulation compared to peripheral blood lymphocytes, and the high expression of IL-4 and IL-10 on intrarenal T cells from LN patients, suggest that intrarenal T cells potentially play a critical role in the pathogenesis of LN (130). Furthermore, CD8^+^ T cells can induce podocyte injury, directly or indirectly, leading to crescent formation (131). Macrophages play a pivotal role in the pathogenesis of LN by mediating impaired kidney repair mechanisms in lupus-susceptible mice. Studies have revealed that these aberrant macrophages facilitate suboptimal kidney repair, precipitating the onset of LN in such mice (132). Similarly, dendritic cells, renowned as the most powerful APCs, are integral to bridging innate and adaptive immunity. Their role in engulfing apoptotic blebs followed by the promotion of Th17 cell differentiation is pivotal, leading to the maladaptive immune responses and the breakdown of self-tolerance that are hallmarks of SLE (133). Furthermore, podocytes are not only structural components of the glomerulus but also actively contribute to immune responses. These cells have been increasingly recognized for their significant role in the pathogenic mechanisms underlying LN. Dysregulated autophagy in podocytes is posited to be a contributory factor in the pathogenesis of SLE (134). Observations indicate that LN stimulates Cyclooxygenase-2(COX-2) in the endoplasmic reticulum stress pathway and activates Activating Transcription Factor 4(ATF4) in podocytes, while inhibition of COX-2 reduces LN-induced autophagy in these cells, highlighting COX-2 as a viable therapeutic target for LN (135). TLR9 has been identified as playing a significant role in the pathophysiology of LN, evidenced by its substantial up-regulation within glomerular structures (136). The interaction between TLR9 and IgG originating from patients is a critical aspect of the disease mechanism. These IgG molecules infiltrate podocytes, eliciting an enhanced expression of calcium/calmodulin-dependent protein kinase IV (CaMK4) (32). This increase in CaMK4 expression subsequently leads to the up-regulation of genes involved in podocyte damage and T-cell activation (137). Moreover, there is a discernible expression and activation of Nod-like receptor protein 3 (NLRP3) inflammasomes within the podocytes in instances of LN, as observed in lupus-susceptible murine models (38). Importantly, the pharmacological inhibition of the NLRP3 inflammasome has been shown to mitigate proteinuria and diminish both the histological deterioration of kidney tissue and the effacement of podocyte foot processes (138). These findings suggest that the activation of NLRP3 plays a crucial role in the advancement of podocyte injury and the ensuing proteinuria, comprising a significant aspect of the pathogenesis of LN.

In recent years, significant progress has been made in understanding the pathogenesis, diagnosis, and treatment of podocyte immune injury in LN. However, there is still a significant research agenda focused on exploring various pathogenic mechanisms and identifying biomarkers associated with this condition. Current research aims to uncover new diagnostic methods and biomarkers to gain a deeper understanding of the underlying processes causing podocyte immune injury in LN. Moreover, identifying potential therapeutic targets is crucial for the development of more effective treatments for this severe complication of SLE.

## 5 Therapeutic strategies targeting immunity in podocyte injury

### 5.1 Current standard therapies for podocyte-related proteinuric glomerular diseases

The clinical manifestations of podocyte-related kidney diseases often present as proteinuria. Most of the diseases are widely acknowledged as immune-mediated kidney disorders. As a result, the standard approach to treating proteinuric glomerular kidney diseases associated with podocyte injury primarily revolves around reducing proteinuria and moderating hyperactive immune responses through the application of immunosuppressive therapy (139). Aside from the commonly used drugs like renin-angiotensin-aldosterone inhibitors, sodium-glucose cotransporter-1 inhibitors, glucagon-like peptide-1 agonists, and mineralocorticoid receptor antagonists, which reduce proteinuria and protect kidney functions through non-immunological mechanisms of action (140), we will focus on the immune-related drugs for podocyte-related proteinuric kidney diseases below.

#### 5.1.1 Immunosuppressive drugs

Glucocorticoids (GCs) have been the mainstay in the treatment of podocyte-related proteinuric kidney diseases for several decades (141). These agents primarily exert their effects by binding to the glucocorticoid receptor (GR) in podocytes, thereby modulating gene expression and influencing the structure and function of these cells (142). For instance, GCs are capable of suppressing the secretion of various pro-inflammatory cytokines, such as interleukins (IL), TGF-β, and tumor necrosis factor (TNF), thereby attenuating the inflammatory response in the glomerulus (143). Furthermore, GCs activate the gene promoter of nephrin, a critical protein in the kidney’s slit diaphragm, thereby facilitating its proper glycosylation and phosphorylation, and reinforcing its connection with the actin cytoskeleton, ultimately maintaining the integrity and stability of the slit diaphragm (144–147). The KDIGO (Kidney Disease: Improving Global Outcomes) 2012 guidelines classified nephrotic syndrome into different types based on the response to glucocorticoid therapy, namely, glucocorticoid-sensitive and glucocorticoid-resistant nephrotic syndrome (148). It has been observed that oral glucocorticoid therapy in children with nephrotic syndrome may encounter issues of resistance, with approximately 50% of affected children eventually progressing to end-stage kidney disease if unresponsive after five years of treatment (149). Consequently, the implementation of specific immunosuppressive therapies, including drugs such as cyclophosphamide, has become necessary (150). The combination of alkylating agents with steroids has demonstrated high efficacy in managing high-risk podocyte-associated proteinuric kidney diseases such as MN (151). Studies have indicated the effectiveness of the combination of cyclophosphamide with prednisone for various high-risk MN patients (152). Research indicates that high-dose cyclophosphamide suppresses CD103^+^ dendritic cells in a rat model, resulting in changes in their frequency, surface molecule expression, and antigen-capturing ability. This alteration enhances CD4^+^ T cell activation, modulates the TLR/MyD88/MAPK pathway, and results in increased Treg levels while reducing Th1/Th2 differentiation and Th17 generation (153). In a ten-year multicenter retrospective study involving 752 pMN patients, cyclophosphamide demonstrated superior performance compared to calcineurin inhibitors. Cyclophosphamide showed statistically significant improvements in treatment response, kidney function preservation, and a lower recurrence rate (154). Despite the fact that calcineurin inhibitors are less effective compared to cyclophosphamide in the management of PMN, they maintain a crucial role in the treatment of podocyte immunological injury.

Calcineurin inhibitors, such as cyclosporine A, tacrolimus, and voclosporin, belong to a class of immunosuppressants that inhibit T cell activation and regulate the Th17 immune response, thereby modulating the occurrence and development of podocyte-related proteinuric kidney diseases (155, 156). Cyclosporine A is of vital importance for the stabilization and protection of podocytes. Evidence has shown that it directly stabilizes the actin cytoskeleton in podocytes, thus helping to maintain the integrity of the glomerular filtration barrier, which is essential for proper podocyte functioning (157). Moreover, research has revealed that Cyclosporine A indirectly safeguards podocytes by regulating the phosphorylation of proteins implicated in the control of actin dynamics, such as WAVE1 (158), and by stabilizing the expression of cofilin-1 (159). Furthermore, Cyclosporine A has proven to be efficacious in addressing specific genetic podocyte damage (160). Tacrolimus is a substance that has been conclusively shown to offer protection to podocytes via mechanisms that include cytoskeleton stabilization, cell apoptosis inhibition, and damage repair (161). Various rodent models of kidney injury have highlighted the ability of Tacrolimus to rejuvenate impaired podocytes and maintain the expression level of Calcineurin Binding Protein 1 (Cabin1) (162, 163). In the context of diabetic nephropathy, Tacrolimus confers protection to podocytes from apoptotic death through downregulating Transient Receptor Potential Channel 6 (TRPC6) (164). Moreover, Tacrolimus aids in mitigating podocytic injury by reinstating FK506 Binding Protein 12 (FKBP12) on the actin cytoskeleton (165). Despite the rapid reduction in proteinuria in pMN upon treatment with calcineurin inhibitors, their long-term effects tend to be unstable, leading to relapses upon discontinuation, and are associated with certain toxic side effects (166). Therefore, the KDIGO 2021 guidelines suggest the use of calcineurin inhibitors in patients with normal kidney function and a moderate risk of disease progression to shorten the duration of proteinuria (139). Voclosporin, a newly developed calcineurin inhibitor, has been licensed in a multitude of countries for the management of lupus nephritis (167). Besides its recognized T-cell immunosuppressive capabilities, Voclosporin serves a dual role in the stabilization of podocytes and exhibiting anti-proteinuric traits. Empirical evidence suggests its superior efficacy in proteinuria reduction, outpacing both cyclosporine A and Tacrolimus (168).

While mycophenolate mofetil (MMF) is well-established as an immunosuppressant for renal immune diseases, the intricacies of its mechanistic effect on podocytes remain to be fully elucidated. Nevertheless, several lines of research provide intriguing insights into potential modes of action. It has been suggested that one facet of MMF’s protective effect against podocyte injury could be attributed to its role in restoring the integrity of the cytoskeletal axis of actin filaments (169, 170). Furthermore, experiments utilizing diabetic mouse models treated with MMF showed a notable reduction in the number of infiltrated CD4 and CD8 T cells within the kidneys. More interestingly, the previous downward trajectory of nephrin and WT1 expression in the glomeruli appeared to be successfully arrested and reversed (171).

While immunosuppressive drugs primarily target immune-mediated glomerular diseases, their use may lead to serious side effects, especially after prolonged use, such as infections, osteoporosis, and hypertension. Moreover, these drugs may not be effective or have limited efficacy in some non-immune proteinuric glomerular diseases, including genetic or metabolic glomerular diseases. Consequently, the development of novel drugs to manage various types of podocyte immune injury is urgently needed.

#### 5.1.2 Biological agents

In recent years, our understanding of the molecular mechanisms and immune targets involved in podocyte injury has advanced, leading to the continuous exploration and development of novel immune-targeted therapeutic strategies (**Table 2**). Biologic agents have emerged as a pivotal treatment option for immune-related diseases and hold significant promise for addressing podocyte-related conditions. RTX, a human-mouse chimeric CD20 monoclonal antibody, disrupts the interaction between B cells and T cells by specifically binding to CD20, inducing CD20^+^ B cell depletion through antibody-dependent and complement-dependent cytotoxic effects (186, 187). In podocytes, RTX also plays a protective role by preserving the actin cytoskeleton, preventing the downregulation of SMPDL-3b, and inhibiting actin cytoskeleton remodeling, ultimately reducing proteinuria (188). A combined treatment approach involving RTX, initial short-term low-dose oral cyclophosphamide, and a rapid tapering of prednisone has shown encouraging results. After a follow-up period of 25 to 62 months for 60 patients with pMN receiving this combined treatment, the study by Zonozi et al. reported that 100% of patients achieved partial remission, with 90% achieving lasting complete remission, while maintaining an acceptable safety profile (189). Ofatumumab (OFA), a new generation of anti-CD20 IgG1κ human monoclonal antibodies, offers an excellent alternative for patients resistant to or sensitized against RTX. OFA is a well-tolerated and safe treatment with longer intervals between doses, which can effectively replace RTX (190). Some studies even suggest that low-dose OFA can induce remission of proteinuria in children with long-term treatment-resistant INS (191). RTX can also be used in combination with calcineurin inhibitors to enhance therapeutic efficacy (192).

**Table 2:** Commonly used immunotherapy drugs for podocyte-related proteinuric kidney disease.

Immunosuppressive drugs	Effect on podocytes	References
Glucocorticoids	Inducing downregulation of pro-inflammatory genes by binding to specific cytoplasmic glucocorticoid receptors (GR); Blocking the TRPC6 signaling pathway and upregulating CD80; Regulating gene expression by binding to glucocorticoid responsive elements (GRE) on DNA; Reducing cell apoptosis by downregulating p53 and increasing miR-30s and Bcl-2	(142, 172–174)
cyclophosphamide	Causing cross-linking of DNA and RNA and inhibiting protein synthesis through cytotoxic effects.	(150)
calcineurin inhibitor	Inhibiting the activation of T cells by targeting the Calcineurin Enzyme while also exerting a direct protective effect on podocytes.	(156, 157)
Calmodulin inhibitors	Preserving the phosphorylation-dependent synaptopodin-14-3-3β interaction; Resulting in upregulation of TRPC6 and ANGPTL4; Reduces cell apoptosis	(175–177)
Mycophenolate mofetil	Reduce uPAR mRNA and protein expression levels; Regulating ATP depletion and stabilizing actin cytoskeleton	(178, 179)
Adrenocorticotropic hormone	Inhibiting cell apoptosis and stabilizing the cytoskeleton	(180)
Rituximab	Binding to CD20 on B cells stabilizes the podocyte cytoskeleton by preventing downregulation of sphingomyelin phosphodiesterase acid-like 3b (SMLPD-3b) and acid sphingomyelinase (ASMase)	(181)
Abatacept	Blocking of B7-1 signal transduction and recovery of SS1 integrin activation	(182)
Eculizumab	Block C5 complement protein and prevent the generation of C5a and C5b	(183)
Avacopan	Inhibits the C5a receptor, preventing the activation of the complement system and the subsequent inflammation and cell damage	(184)
Narsolimab	Inhibits the MASP-2, thus preventing the activation of the complement system	(185)

Abatacept, categorized as an immunomodulatory drug, is formed by fusing cytotoxic T-lymphocyte-associated antigen 4 (CTLA-4), a surface protein, with the Fc segment of human immunoglobulin. External factors like viral infections and Treg function can impact the expression of B7-1 (CD80) on podocytes, potentially causing podocyte cytoskeletal disarray and severe proteinuria (193). CTLA-4, a critical co-factor expressed on podocytes and Tregs, binds to B7-1 (CD80), inhibits T cell activation, and reinstates SS1 integrin activation, thereby alleviating podocyte injury and proteinuria (194). Contrary to conventional wisdom, recent research challenges the notion of increased B7-1 expression within the podocytes of individuals with proteinuria. Studies investigating minimal change disease (MCD) and focal segmental glomerulosclerosis (FSGS) utilizing multiple antibodies and assays found no significant upregulation of podocyte B7-1 in these conditions, casting doubt on the efficacy of B7-1 inhibitory treatment (195). Moreover, B7-1 is not induced in podocytes of patients with diabetic nephropathy (DN) or in BTBR ob/ob mice, a type 2 diabetes model (196). A recent study exploring abatacept’s efficacy in post-kidney transplant FSGS patients who developed FSGS after transplantation and failed conventional therapy revealed that responders to abatacept were B7-1 positive. This suggests that podocyte B7-1 staining in kidney transplant biopsies might identify patients benefiting from abatacept (197). Researchers propose that the mechanism behind the anti-proteinuric effects of B7-1 inhibitors may involve the suppression of immune cell activation rather than direct effects on podocytes (195). Furthermore, some evidence suggests a direct podocyte protective role of abatacept (198). Consequently, further research is crucial to elucidate the association between B7-1 and podocyte damage in different kidney conditions.

Immunotherapy, which leverages the immune system to intervene in podocyte damage, is a promising approach known for its high specificity, minimal side effects, and durable benefits. It can be applied to treat various types of podocyte damage. Future research should prioritize a deeper understanding of the mechanisms involved and the optimization of parameters for immunotherapy to improve its clinical applicability and effectiveness.

### 5.2 Novel therapeutic approaches

In recent years, as our understanding of the immune mechanisms underlying podocyte injury in proteinuric kidney diseases has deepened, new prospects for immunotherapy in managing podocyte diseases have emerged. The involvement of the complement system in the pathogenesis of podocyte injury has been highlighted (199). C5b is known to induce osmotic lysis of podocytes, leading to alterations in the actin cytoskeleton and the podocyte slit diaphragm, as well as limiting the proliferation of podocytes (200). Therefore, the utilization of the complement inhibitor Eculizumab to impede the activation of the complement cascade and the subsequent generation of C5b constitutes an effective strategy for intervention (183). Avacopan, as a selective inhibitor of the C5a receptor, orchestrates a blockade against C5a activity (201). By attenuating the complement system’s activation and thereby abating subsequent inflammatory responses and cellular damage, this inhibition facilitates an indirect protective effect on podocytes through the modulation of the inflammatory environment (184). Narsolimab, or OMS721, is a fully human monoclonal antibody that specifically targets and inhibits mannan-binding lectin-associated serine protease-2 (MASP-2) (202). MASP-2 serves as the effector enzyme within the lectin pathway of the complement system, which is regarded as one of the principal routes of complement activation (203). By effectively inhibiting MASP-2, narsolimab disrupts the activation of the lectin pathway, thereby mitigating complement-mediated inflammation and minimizing endothelial damage (204). In a recent study, the administration of narsolimab demonstrated clinically significant reductions in proteinuria and sustained stability in estimated glomerular filtration rates (185).

In the case of patients with MCD, podocytes express angiopoietin-like 4 (Angptl4), with one of its forms being hyposialylated Angptl4, which binds to the glomerular basement membrane and endothelial cells, leading to proteinuria (205). Utilizing N-acetyl-D-mannosamine has shown promise in converting hyposialylated Angptl4 to its sialylated form, which can be taken up and stored by podocytes, thereby reducing the production of proteinuria (206). Although this appears to be a potential treatment option, relevant clinical data remains limited.

The field of immunotherapy for podocyte preservation is in a state of constant advancement, highlighted by the emergence of various natural compounds and small-molecule inhibitors known for their podocyte-protective properties. For example, curcumin, derived from turmeric, has been recognized for effectively reducing reactive oxygen species (ROS) and RIPK3 expression, thereby establishing itself as a potential therapeutic agent for combating diabetic nephropathy (207). Additionally, luteolin exerts a protective effect against glucose-induced podocyte stress by inhibiting the NLRP3 inflammasome, a critical component of the immune system and inflammatory response (208). Expanding the scope, Astragaloside IV is acknowledged for its comprehensive protective capabilities, which include mitigating endoplasmic reticulum stress, promoting autophagy, and improving mitochondrial function (209). This multitargeted approach involves interaction with several signaling pathways such as SERCA2, AMPKα, and Nrf2-ARE/TFAM, highlighting the molecule’s potential in supporting podocyte integrity (210, 211). In a similar vein, hyperoside presents a protective strategy by modulating mitochondrial dynamics. It specifically inhibits mitochondrial fission, which has been observed to diminish albuminuria and renal damage in experimental models, further emphasizing its therapeutic potential (212).

Within the realm of metabolic regulation, the Rho-associated coiled-coil-containing protein kinase 2 (ROCK2) signaling pathway has been identified as a crucial regulator with significant implications for podocyte health. The ROCK2 signaling pathway plays an instrumental role in numerous metabolic functions critical to podocyte health, notably the initiation of apoptosis (213). Additionally, it disrupts fatty acid oxidation through the downregulation of peroxisome proliferator-activated receptor alpha (PPARα), thus underlining the pathway’s fundamental influence on cellular metabolism and the podocyte’s adaptability to metabolic stress (214). Additionally, in the intricate web of intracellular signaling, the mechanistic target of the rapamycin (mTOR) pathway stands out, particularly through its two distinct complexes, mTORC1, and mTORC2, as a vital target for immunotherapeutic intervention (215). Inhibiting these complexes has been shown to offer reprieve to stressed podocytes, principally by counteracting the adverse effects of hyperglycemia (216). These effects extend to safeguarding cell viability, reducing apoptosis, and maintaining the stability of structural proteins, which are fundamental to podocyte function. Moreover, contemporary research has shed light on the utility of angiotensin II inhibition in podocyte protection and the retardation of glomerulosclerosis progression (217). The benefits of this inhibition extend beyond the direct antagonism of angiotensin II type 1 (AT1) receptors on the podocytes themselves (218). Emerging evidence suggests additional protective mechanisms that may involve crosstalk with other cellular entities or alternative molecular pathways, presenting potential avenues for therapeutic interventions (**Table 3**).

**Table 3:** Some natural medications with immunotherapeutic effects on podocyte injury.

Nature medicine	Effect on podocytes	References
Curcumin	Attenuates Angiotensin II-induced podocyte injury and apoptosis by inhibiting endoplasmic reticulum stress; Reduce the expression of ROS and RIPK3	(207, 219)
Luteolin	Attenuates high glucose-induced podocyte injury via suppressing NLRP3 inflammasome pathway	(208)
Astragaloside IV	Collaborate with SERCA2 and AMPK α Multiple signaling pathways such as Nrf2-ARE/TFAM protect podocytes	(210, 211)
Hyperoside	Reduces albuminuria in diabetic nephropathy at the early stage through ameliorating renal damage and podocyte injury	(220)

Collectively, these diverse strategies underscore the rapid advancement in the realm of podocyte immunotherapy, paving the way for novel, tailored treatment options aimed at reducing the burden of podocyte injury and related kidney diseases. Each compound or pathway presents its mechanism of action, which when considered in conjunction, could offer a comprehensive, multi-targeted approach to combat renal pathologies associated with podocyte damage.

## 6 Conclusion remarks

Podocytes play a crucial role in both renal physiology and pathology, providing valuable insights into kidney function. While prior research has highlighted tubulointerstitial fibrosis as a key pathological mechanism in the progression and kidney failure of CKD, recent studies have also underscored the connection between the dysfunction and death of podocytes and the advancement of CKD. The immune response of podocytes serves as a pivotal factor in the development and progression of various proteinuric kidney diseases and presents itself as a promising therapeutic target. Despite advancements, numerous aspects of the mechanisms governing podocyte immune responses remain uncharted, emphasizing the need for further exploration. Subsequent research endeavors should aim to identify novel podocyte-related antigens and antibodies, unravel the intricacies of the regulatory mechanisms governing the interaction between podocytes and other immune cells, and develop innovative therapeutic strategies that specifically target podocyte immune responses. These efforts are essential for enhancing the prognosis and overall quality of life for patients affected by kidney disease and for addressing the current unmet medical needs in this area.

## Author contributions

HJ: Conceptualization, Supervision, Writing – review & editing. ZS: Investigation, Writing – original draft. JZ: Investigation, Writing – review & editing. CL: Funding acquisition, Writing – review & editing. YQ: Writing – review & editing. CX: Software, Writing – review & editing. SY: Writing – review & editing. XT: Conceptualization, Supervision, Writing – review & editing.
